# Enhanced Sensing Capacity of Terahertz Triple-Band Metamaterials Absorber Based on Pythagorean Fractal Geometry

**DOI:** 10.3390/ma15186364

**Published:** 2022-09-13

**Authors:** Alin Gheorghita Mazare, Yadgar I. Abdulkarim, Ayoub Sabir Karim, Mehmet Bakır, Mohamed Taouzari, Fahmi F. Muhammadsharif, Bhargav Appasani, Olcay Altıntaş, Muharrem Karaaslan, Nicu Bizon

**Affiliations:** 1 Faculty of Electronics, Communication and Computers, University of Pitesti, 110040 Pitesti, Romania; 2 Medical Physics Department, College of Medicals & Applied Science, Charmo University, Chamchamal 46023, Iraq; 3 Physics Department, College of Education, Salahaddin University-Erbil, Erbil 44002, Iraq; 4 Department of Computer Engineering, Bozok University, Yozgat 66200, Turkey; 5 Laboratory LISA, Hassan First University of Settat, National School of Applied Sciences, Berrechid 26100, Morocco; 6 Department of Physics, Faculty of Science and Health, Koya University, Koya 44023, Iraq; 7 School of Electronics Engineering, KIIT University, Bhubaneswar 751024, Odisha, India; 8 Department of Electrical-Electronics Engineering, Iskenderun Technical University, Hatay 31200, Turkey; 9 Doctoral School, Polytechnic University of Bucharest, 313 Splaiul Independentei, 060042 Bucharest, Romania; 10 ICSI Energy Department, National Research and Development Institute for Cryogenic and Isotopic Technologies, 240050 Ramnicu Valcea, Romania

**Keywords:** metamaterials (MTM), THz, sensor, perfect absorber

## Abstract

A new design of a triple band perfect metamaterial absorber based on Pythagorean fractal geometry is proposed and analyzed for terahertz sensing applications. The proposed design showed an enhanced sensing performance and achieved three intensive peaks at 33.93, 36.27, and 38.39 THz, corresponding to the absorptivity of 98.5%, 99.3%, and 99.6%, respectively. Due to the symmetrical nature of the recommended design, the structure exhibited the characteristics of independency on the incident wave angles. Furthermore, a parametric study was performed to show the effects of the change in substrate type, resonator material, and substrate thickness on the absorption spectrum. At a fixed analyte thickness (0.5 μm), the resonance frequency of the design was found to be sensitive to the refractive index of the surrounding medium. The proposed design presented three ultra-sensitive responses of 1730, 1590, and 2050 GHz/RIU with the figure of merit (FoM) of 3.20, 1.54, and 4.28, respectively, when the refractive index was changed from 1.0 to 1.4. Additionally, the metamaterial sensor showed a sensitivity of 1230, 2270, and 1580 GHz/μm at the three resonance frequencies, respectively, when it was utilized for the detection of thickness variation at a fixed analyte refractive index (RI) of 1.4. As long as the RI of the biomedical samples is between 1.3 and 1.4, the proposed sensor can be used for biomedical applications.

## 1. Introduction

Terahertz (THz) waves ranging from 0.1 to 40 THz have several applications in THz imaging [1,2,3], energy harvesting [4,5,6,7], and wireless communications [8,9,10,11]. Metamaterials, which are artificial structures with periodic or non-periodic sub-wavelength designs in micrometer dimensions, enable the controlling and manipulating of electromagnetic waves [12]. The fact that THz radiation can be absorbed by metamaterials makes them very useful in a wide range of applications. The state-of-the-art THz metamaterial absorbers are characterized by a wide bandwidth [13], multiple resonance response [14], and narrow bandwidth [15]. An increase in the bandwidth can be realized by combining sub-unit cells [16,17], stacking metal–dielectric–metal layers [18], or using composite structures such as graphene or doped silicon [19,20]. Fractal geometry is widely used to achieve broadband absorption with different metal–dielectric–metal combinations [21,22,23,24]. Bilal et al. demonstrated a wideband absorption in the terahertz spectrum ranging from 7.5 to 10 THz [25].

Metamaterial absorber-based high-sensitivity THz sensors have drawn attention in recent years [26,27,28]. The resonance peak frequency is sensitive to the refractive index of the test medium (analyte), which can be used to develop a highly sensitive THz sensor [29]. Zhou et al. [30] designed a THz biosensor that significantly improved the sensitivity by enlarging the overlap. Chen et al. [31] used a 3D graphene-based meta-structure to achieve high sensitivity in sensing the refractive index. Another refractive index-based terahertz sensor was proposed by Veeraselvam et al. [32], which showed a peak sensitivity of 22.75 GHz/RIU for various sample loadings. Yao et al. proposed a metamaterial THz sensor with a flexible substrate [33]. Their simulation studies showed a refractive index sensitivity of 60 and 100 GHz/RIU at two respective resonance peaks. Another metamaterial absorber-based THz sensor was realized by Saadeldin et al. [34] that can be used as a refractive index sensor with a high sensitivity of 300 GHz/RIU and the figure of merit (FoM) of 2.94 in the RI range from 1.0 to 1.39 at the analyte thickness of 1.0 μm. A dual-band terahertz metamaterial absorber composed of two identical square metallic patches was demonstrated by Wang et al. [35]. Resonance peaks with near-unity absorption were obtained with a high sensing sensitivity of 1.9 THz/RIU.

Although different structures have been proposed to achieve multiband or wideband absorption with sensor applications, there are still shortcomings, such as complex design and narrow bandwidth [26,27,28,29,30,31,32,33,34,35,36,37,38,39,40]. The majority of the previously reported designs are hard to fabricate, which means they are complex structures. On the other hand, the reported designs generally suffer from showing a wide bandwidth. Therefore, in the current work, a simple design with a low-profile structure was proposed. Additionally, the ZnSe substrate was utilized due to its low electrical resistivity, high direct band gap, large refractive index, low optical absorption, great photosensitivity, and wide transparency over a wide frequency range. We believe that our proposed design can be potentially applicable in the field of stealth technology, imaging, and thermal energy harvesting.

This paper proposes a Pythagorean-tree-based fractal geometry for wideband absorption and sensing applications. The proposed structure can be effectively used in refractive index sensing between 30 THz and 40 THz. Due to the complexity of fabrication, previous reported simulated works are hard to validate with experimental results.

The rest of the present manuscript is organized as follows. The next section describes the design and the steps involved in arriving at the design. In the third section, the sensing performance of the design is presented. The theoretical explanation of the absorption mechanism is given in section four, while the last section summarizes the work.

## 2. Design of the Triple-Band MTM Perfect Absorber

### 2.1. Structure Optimization and Boundary Conditions

**Figure 1:**
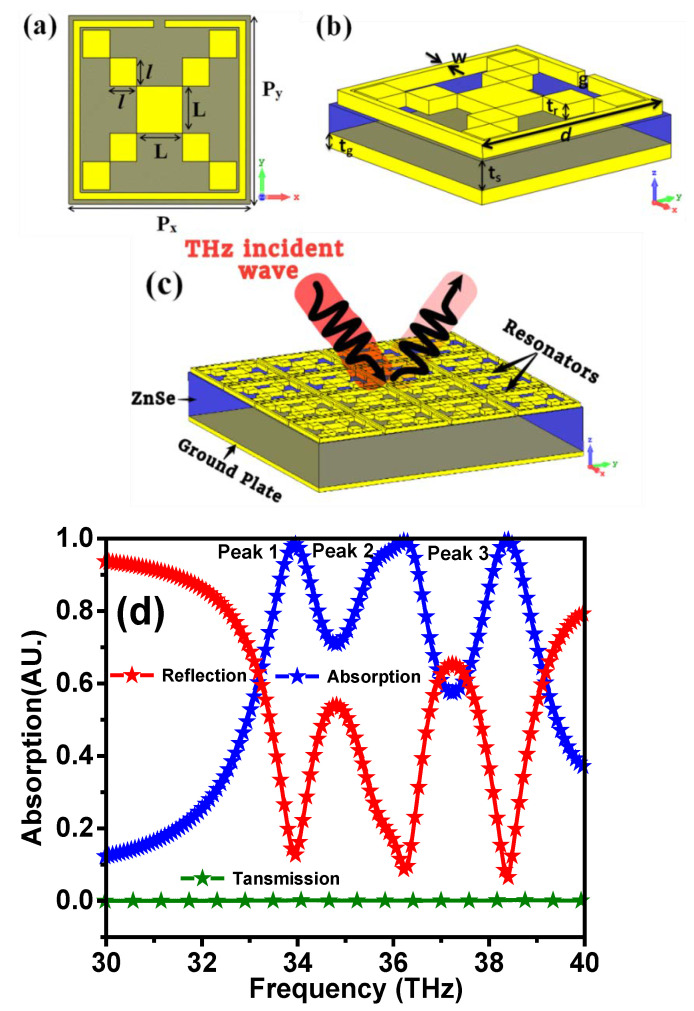
(**a**) Top view of the proposed design, (**b**) perspective view, (**c**) the 4 × 4 array unit cell showed all layers with incident THz, and (**d**) simulated absorption, reflection, and transmission spectra.

The schematic diagram of the metamaterial (MTM) design consisting of a Pythagorean fractal geometry loaded with a complimentary quadratic split ring resonator on top of a ZnSe substrate is shown in Figure 1. The finite integration technique of the Computer Simulation Technology (CST) microwave studio 2018 software was used to design and obtain the numerical results. Several boundary conditions were used in the numerical software according to the applications used. In this design, periodic boundary conditions were set along the *x*- and *y*-axis, while the plane wave is incident along the *z*-axis. The impedance matching between the resonator and free space can be obtained by adjusting the design parameters. This reduces the wave reflection and increases the absorption of the incident wave. The parametric dimensions of the proposed design were purposely optimized by using the genetic algorithm (GA) optimization technique provided in the CST software. The optimized conditions were utilized to simulate the triple bands of perfect absorption in the terahertz frequency range. The optimized parameters are shown in Table 1.

The top and bottom metallic layers were made of aluminum with a conductivity of 5.96 × 10^7^ S/m. The top layer has a thickness of *t_r_* = 0.03 μm, and the bottom layer thickness is *t_g_* = 0.03 μm. The dielectric layer in the middle is made of ZnSe with *t_s_* = 0.6 μm. The square patch has a side length of L = 2 μm, and the other square patches have a side length of l = 1.2 μm. The outer split ring has a side length of d = 7.6 μm, split gap of g = 0.5 μm, and width of w = 0.3 μm. The unit cell dimension is P_x_ = 8 μm and P_y_ = 8 μm. The parameters of each material in the design were taken from the material library of the simulation software (CST). Figure 1b,c shows the perspective view and the 4 × 4 array unit cell of the proposed structure that shows all layers with a THz incident wave. Figure 1d shows the numerical results of the absorption, reflection, and transmission for the proposed metamaterial structure in the frequency range from 30 to 40 THz. It can be seen from Figure 1d that there are three intensive peaks of more than 98% absorptivity at 33.93, 36.27, and 38.39 THz. In this structure, a metallic layer made of aluminum was purposely used at the bottom of the structure to block the electromagnetic waves, resulting in the minimized reflection coefficient and, hence, achieving perfect absorption. On the other hand, the bottom aluminum plane was taken to be sufficiently thick (with a thickness greater than its skin depth) in order to obtain a zero transmission. However, the thickness of the top plane should be less than the skin depth so that it allows the wave transmission to reach the dielectric layer. By using the equation below, the absorptivity of the proposed design can be obtained [40].

(1) A(w)=1−R(w)−T(w)

where $T\left(w\right)={\left|S_{21}\right|}^{2}$ and $R\left(w\right)={\left|S_{11}\right|}^{2}$, *A(ω)* are the transmission, reflection, and absorption coefficients, respectively.

### 2.2. The Step-by-Step Layout of the Proposed Metamaterial Unit Cell

**Figure 2:**
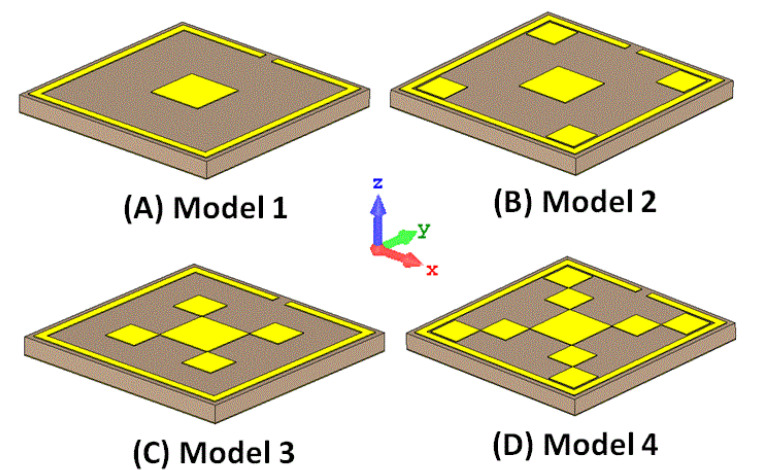
Steps involved in the design of the proposed absorber.

**Figure 3:**
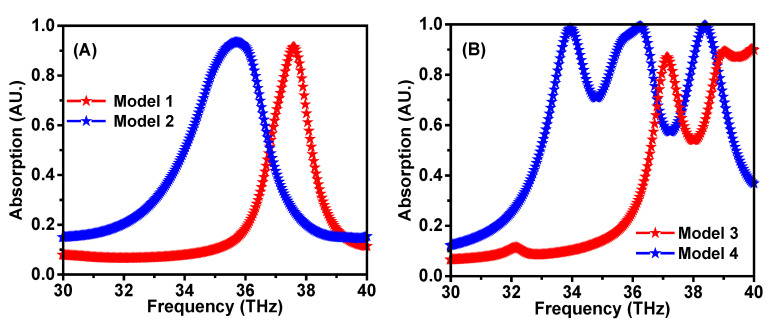
Absorption spectra of Model 1 and 2 (**A**) and Model 3 and 4 (**B**).

Figure 2 shows the schematic view of the step-by-step of the proposed absorber design, which consists of a Pythagorean fractal geometry loaded with a complimentary quadratic split ring resonator on top of the ZnSe substrate. The proposed structure is made of Al, ZnSe, and Al layers, respectively. This design was specifically chosen because of its easy production. The proposed design was realized in four steps by analyzing four different models, as shown in Figure 2. In the first step, a square patch was placed in the middle of a split ring resonator (Model 1). In the next step, four squares were placed at the inner end of the split ring, as shown in Model 2. In the third step, the middle square patch was surrounded by four identical square patches positioned at the corners of the center patch (Model 3). As such, Model 4 was assigned to be the proposed design. The four-unit cells were designed and simulated in CST Microwave Studio. A plane electromagnetic wave was incident on the unit cells, with periodic boundary conditions along the edges. The absorption spectra of the four models were recorded, as shown in Figure 3. The significance of choosing the frequency range from 30 to 40 THz is to make the proposed structure applicable for the refractive index sensing, stealth technology, imaging, materials detection, active THz modulator, and switcher.

**Figure 4:**
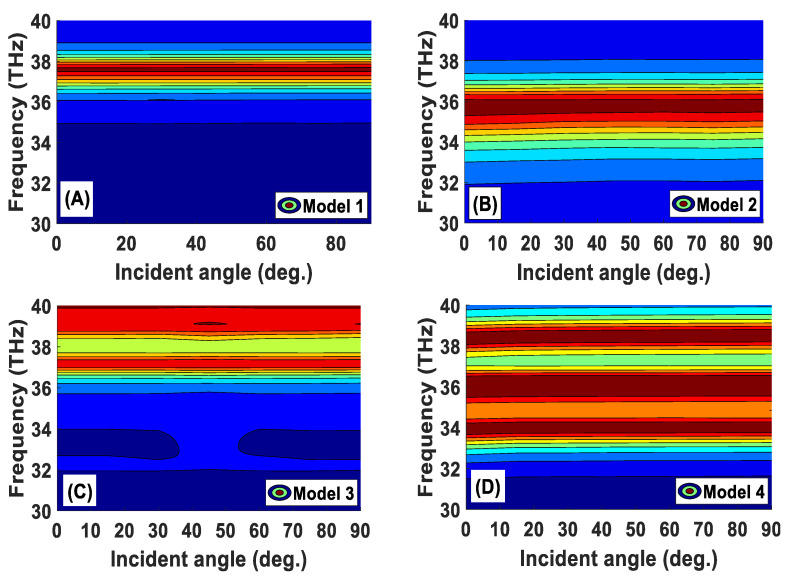
Color contour plot of frequency-dependent incident angle for different proposed models. The color bar on the right side of the figure shows the absorption value.

In Model 1, the fractal stage was realized using a single *L* × *L* square loaded complementary quadratic split ring resonator. Figure 3A depicts the absorption characteristic of Model 1. The absorber exhibited more than 80% of absorption in the frequency range from 37.27 to 37.88 THz, and the absorption peak was observed at 37.58 THz. It can be seen from Figure 4A that the same absorption results were achieved for the incident angles ranging from 0 to 90 degrees. Figure 3A shows the absorption response of Model 2, which showed more than 80% of absorption from 35 to 36.5 THz, with a peak of absorption at 35.8 THz. Then, Model 3 is proposed to increase the absorption range. Figure 3B depicts the absorption response of Model 3, in which the absorption peaks were observed at 37.16 and 39.05 THz. The absorption performance of the final stage (Model 4) is depicted in Figure 3B, which shows three absorption peaks at 33.93, 36.27, and 38.39 THz. As shown in Figure 4, the absorption findings are stable for the incident angles ranging from 0 to 90 degrees [41]. The proposed metamaterials absorber has potential applications in refractive index sensing, stealth technology, imaging, material detecting, active THz modulator, and switcher. The designed structure has only three perfect absorption peaks in the frequency range, which can be specifically useful for biomedical imaging applications. The authors have designed and optimized this structure to be compatible with the THz frequency. Changing the lateral dimensions of the structure does not affect the resonance frequency positions but affects the absorption response.

Figure 4 shows the color contour plot of the four models of the metamaterials absorber in the incident angle range from 0 to 90°. Noticeably, a single sharp peak was seen at about 37.6 THz for Model 1, where the horizontal brown-colored bar represents its peak position. Nevertheless, the position of this peak was found to be red-shifted in Model 2, yet with a larger band of absorption as the brown bundle has become larger (see Figure 4B). Also, two and three main peaks were observed for Model 3 and Model 4, respectively. Noteworthy, the red-colored bands for Model 3 are less intensive compared to those of Model 4, in which three major brown-colored bundles can be seen. Noticeably, no significant variation was observed in the thickness and color of the horizontal bars that correspond to the absorption peaks. This indicates that the position and value of the absorption peaks remained unchanged with the changes in the incident angle.

### 2.3. Pythagorean Fractal Resonance in a Wide Frequency Range

In order to obtain an ideal absorber unit cell, attempts were made to obtain a triple band metamaterial absorber with the lowest reflectance and transmittance responses. Hence, the observations are needed to choose the best parameters needed for designing the structure so that a wide absorption band is achieved. It was observed that by choosing 0.6 µm thick of ZnSe, copper metal, for the top and bottom layer, the best absorption characteristics were achieved.

**Figure 5:**
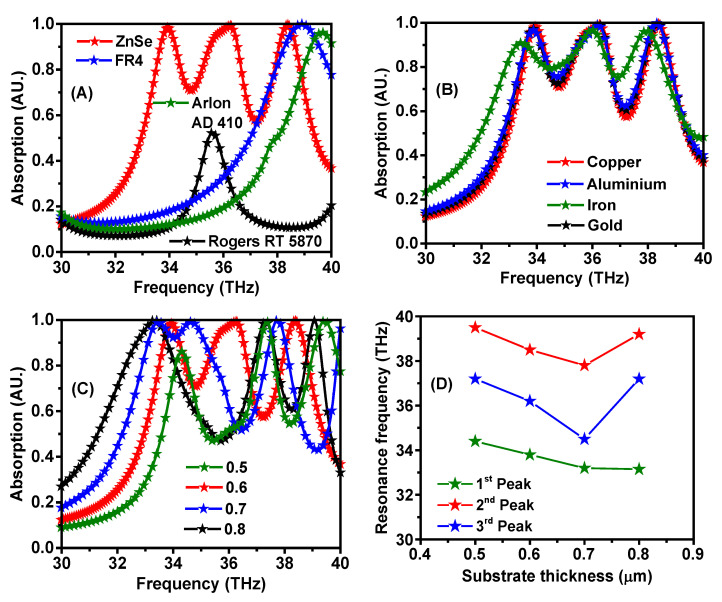
(**A**) Parametric study for different substrate materials, (**B**) various resonator materials, (**C**) change of the thickness of the substrate, and (**D**) substrate thickness dependence of the resonance frequency shift.

In order to optimize the absorber unit cell towards enhancing the sensing capacity of the triple-band metamaterial, different substrate types were used, including ZnSe, FR4, Arlon AD410, and Rogers RT 5870. To obtain ideal resonators, four different design models were proposed with test effects of different substrate thicknesses, as shown in Figure 5. One can see from the figure that by using FR4, Arlon AD410, and Rogers RT 5870, a single resonance peak resulted, with a strong absorption band for FR4, Arlon AD 410 substrates at 38.4, 39.5, and 35.5 THz, respectively, while a weak absorption band was observed for Rogers RT 5870. However, by utilizing the ZnSe substrate, a strong triple-band absorption was achieved at the resonance frequencies of 33.8, 36.3, and 38.1 THz, as shown in Figure 5A. In these investigations, the thickness of the substrate and resonator were fixed at 0.6 and 0.03 μm, respectively. On the other hand, by considering the ZnSe substrate and using copper, aluminum, and gold resonators, the highest absorption values were recorded at 33.8, 36.1, and 38.1 THz, respectively, which were found to be higher than that of the iron resonator, as shown in Figure 5B. Further attempts were made to study the effects of changing thickness values of the ZnSe substrate on improving the absorption properties, as shown in Figure 5C. Noticeably, there is a triple-band absorption at different resonance frequencies, which was obtained over a wide range of substrate thicknesses. However, different thicknesses act to maximize the energy of absorbance and its properties, as shown by the offsets of the resonance frequency peaks in Figure 5D.

## 3. Improved Sensing Performance

**Figure 6:**
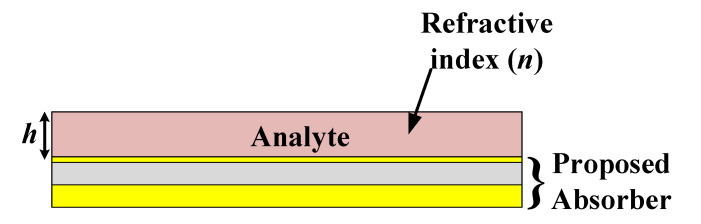
Proposed absorber as a dual sensor for sensing refractive index of the analyte and its height.

It is noteworthy that the designed sensor can act as a dual sensor. It can sense both the refractive index of the analyte and its height. This can be explained with the help of the diagram illustrated in Figure 6. The height of the analyte in the simulation software was fixed at 0.1 μm for the refractive index sensing, while the value of the refractive index was fixed at 1.4 for the analyte sensing performance.

**Figure 7:**
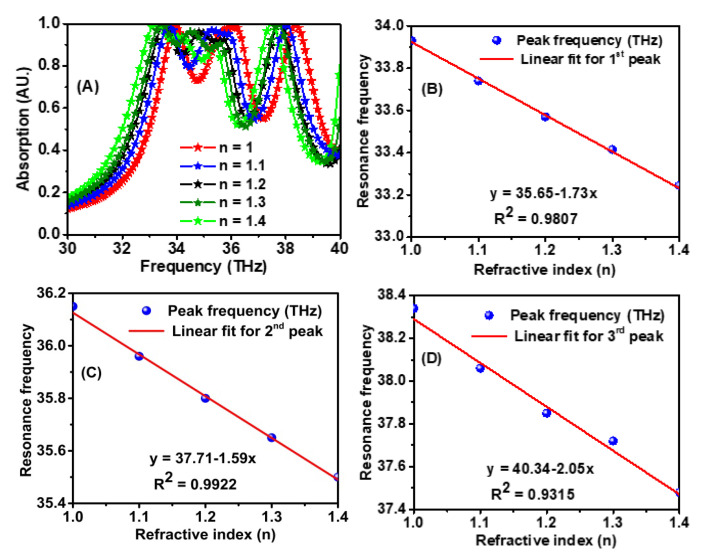
(**A**) Absorption spectrum dependence on the variation of the refractive index of the surrounding, and (**B**–**D**) shows the resonance frequency peak with the linear fit of the three frequencies versus the refractive index.

The refractive index of the analyte varied between 1 and 1.4, while the analyte thickness was fixed at 0.1 μm, and the corresponding absorption spectra were plotted, as shown in Figure 7A. Results showed that the resonance peaks shifted to the left with the increase in the refractive index. The resonance frequency versus the refractive index for each resonance peak is shown in Figure 7B–D. The sensitivity for these peaks is 1.73, 1.59, and 2.05 THz/RIU, respectively.

**Figure 8:**
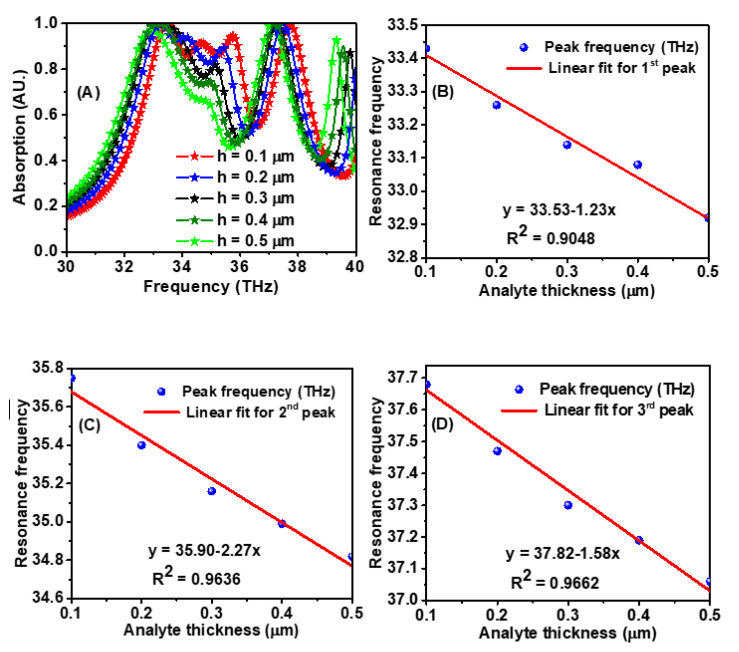
(**A**) Absorption spectrum dependence on the variation of analyte depth and (**B**–**D**) shows the frequency shift versus different analyte depths for the three resonance frequencies.

In the next part of the investigations, the refractive index of the analyte was fixed at 1.4, and its height was varied. The absorption spectra at different analyte heights and a refractive index of 1.4 are given in Figure 8A. When the depth of the analyte was increased, the absorption spectrum shifted to the left. The resonance frequency versus the analyte depth is shown in Figure 8B–D. The sensitivity of the sensor towards the analyte depth is 1.23 THz/μm in the first resonance band, 2.27 THz/μm in the second resonance band, and 1.58 THz/μm in the third resonance band. Thus, the proposed design can sense both the analyte thickness and its refractive index and, hence, can find its application in biomedical sensing.

The figure of merit “FOM” is another important parameter used to compare the sensing performance of various sensors, which is defined as the ratio of sensitivity to the full width at half maximum “FWHM” [34]:

$$\mathrm{FOM}=\frac{S}{\mathrm{FWHM}}$$

We calculated the figure of merit “FOM” of the three resonance peaks with varied thicknesses of the analyte layer, and it was found to be 3.20, 1.54, and 4.28 for the 1st, 2nd, and 3rd peaks, respectively.

## 4. Theoretical Explanation for Enhanced Sensing Performance

In this section, surface current and electric field distributions were examined to observe the enhancement during the evolution process of the proposed structure. Moreover, it is important to understand the working mechanism and physical behavior of the structure in terms of these distributions. The field distributions were observed for all the models given in Figure 2, especially at their resonance frequencies.

**Figure 9:**
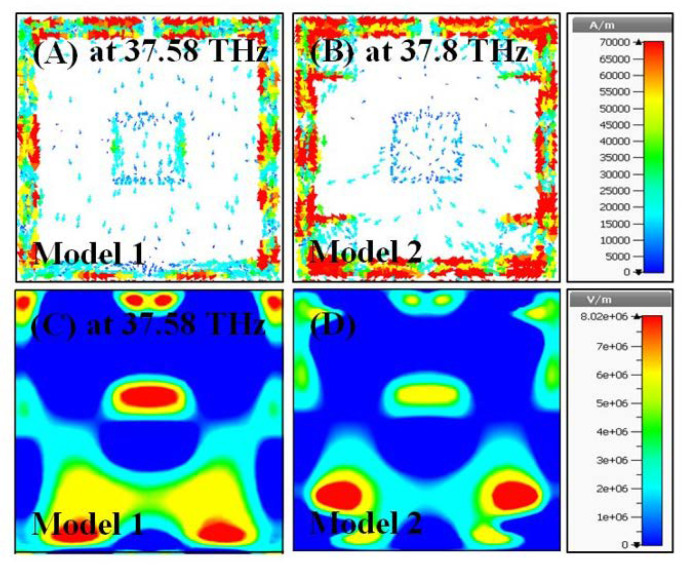
Color map of the field distributions for Model 1 and Model 2 (**A**) surface current at 37.58 THz for Model 1, (**B**) surface current at 37.8 THz for Model 2, (**C**) electric field at 37.58 THz for Model 1, (**D**) electric field at 37.8 THz for Model 2.

The surface current distributions for Model 1 and 2 particularly occurred around the SRR at 37.58 THz and 37.8 THz, respectively, as illustrated in Figure 9A,B. The electric field distribution for Model 1 is slightly intense compared to that of Model 2 at the mentioned frequencies, as shown in Figure 9C,D. The reason can be explained by the surface current distribution behaviors of the models. As the surface current in Model 2 distributes a bit more than in Model 1, the impedance matching, which causes the influencing ratio of the electromagnetic wave, becomes lower for Model 2. Hence, the electric field strength is decreased relatively for Model 2.

**Figure 10:**
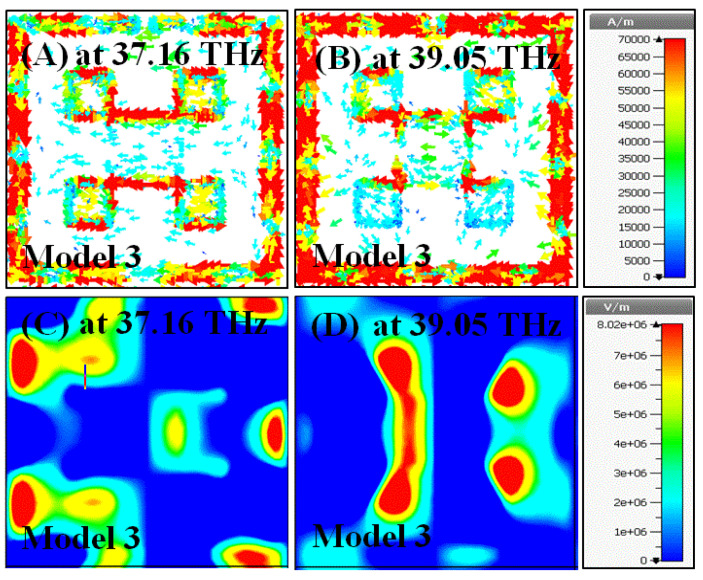
Simulated color map of the field distributions of Model 3 (**A**) surface current at 37.16 THz, (**B**) surface current at 39.05 THz, (**C**) electric field at 37.16 THz, (**D**) electric field at 39.05 THz.

In Figure 10, the field distributions have been investigated for Model 3 at two different resonance frequencies of 37.16 THz and 39.05 THz. The surface current was relatively concentrated in the middle of the resonator at 37.16 THz, while it was distributed around the split ring part of the resonator at 39.05 THz, as illustrated in Figure 10A,B. In comparison to the surface current distributions, the electric field is stronger at the corner region for Model 3 at 37.16 THz, and it is concentrated in the middle at 39.05 THz, as demonstrated in Figure 10C,D. The density of the surface currents at the resonance frequencies makes the impedance matching between the free space and the proposed structure weaker. Hence, the absorption ratio for Model 3 is lower than the other models illustrated in Figure 3.

**Figure 11:**
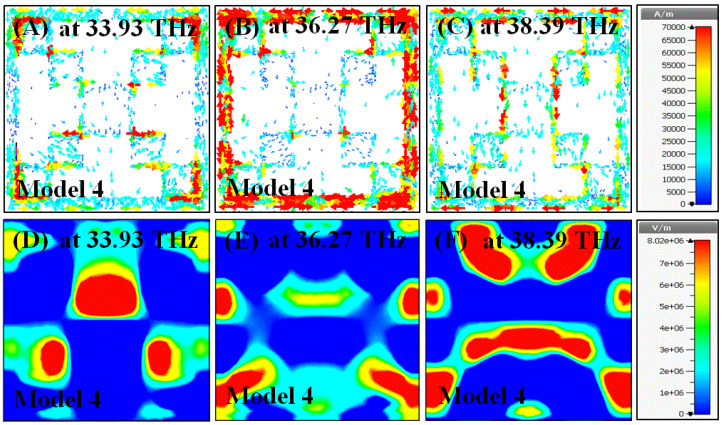
Color map of the current and electric field distributions of proposed metamaterial structure surface current and electric field at three different frequencies.

The surface current and electric field distributions of the proposed metamaterial were investigated at three resonance frequencies of 33.93, 36.27, and 38.39 THz, as shown in Figure 11. The surface currents were highly distributed on the corners at 33.93 THz, around the split ring at 36.27 THz, and homogenously at 38.39 THz, as illustrated in Figure 11A–C, respectively. The electric fields were much stronger in the middle at 33.93 THz, at the corners at 36.27 THz, and in both the middle and corners at 38.39 THz, as illustrated in Figure 11D–F, respectively. The proposed model has the feature of the lowest surface current distribution and the most intense electric field concentration at the resonance frequencies compared to those of the other models. Hence, it can be said that it has the best impedance matching performance with a triple-band characteristic.

**Figure 12:**
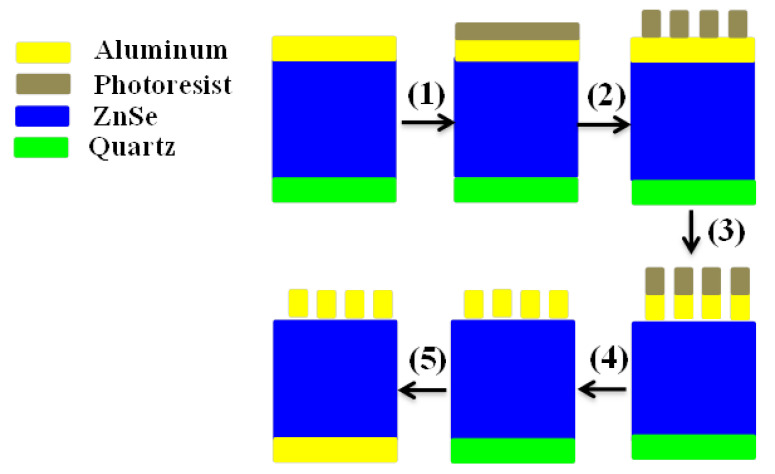
Process flow: (1) and (2) is the photolithography to define the metamaterial geometry, while (3) and (4) show the wet etching and washing of the photoresist to fabricate the metamaterial structures, and (5) is the evaporation of metallic substrate layer on another side of the ZnSe layer.

As shown in Figure 12, the proposed design can be manufactured by using a 10 μm thick of ZnSe layer with a 2 μm coated aluminum film. Ethanol and acetone were first used to clean a 3 mm quartz wafer. Tweezers were then used to delicately lay the cleaned ZnSe-aluminum film onto the wafer’s surface. Because acetone volatilizes quickly, pressing the two layers rapidly and accurately was required to minimize air between the substance and the wafer surface. The substance appeared to be adsorbed to the wafer due to the comparatively flat surface. The sample was then spin-coated with positive liquid photoresist RZJ-304 and baked for 90 s at 100 °C. Finally, the sample was moved to the photolithography processing machine, where a 60 × 60 mm^2^ array was fabricated. The part of the altered photoresist was washed with a developer for 30 s after processing. The exposed metal was then etched with aluminum. The metamaterial structures were obtained as a result. Finally, acetone was used to clean the sample and remove the remaining photoresist. Consequently, a 2 μm thick metallic aluminum layer was produced on the other side of the ZnSe layer using a vacuum evaporation process. A metamaterial of 60 × 60 mm^2^ periodic metallic arrays with a dimension of 9 × 9 mm^2^ was produced. Terahertz time-domain spectroscopy was used to characterize the reflection spectra. The periodic boundary condition was addressed while the sample was irradiated by a light source with a spot diameter of 6 mm.

Table 2 shows the comparison of the overall performance of the proposed MTM structure with that of other works reported in the literature. Comparatively, it can be seen from Table 2 that, based on the techniques of the MTM structure, unit cell size, thickness of the used substrate, the figure of merit, and sensitivity. The proposed design has a smaller unit cell of 8 × 8 μm^2^, processes an ultra-thin property of 0.6 μm, and higher sensitivity compared to those of other designs reported in the literature.

## 5. Summary

The design and analyses of a new perfect metamaterial absorber were successfully achieved by incorporating a Pythagorean fractal geometry resonator. The device can be used for sensing applications in the terahertz frequency range. The results showed three high absorption peaks of more than 98% absorptivity at 33.93, 36.27, and 38.39 THz. The designed structure showed excellent sensitivity of 1730, 1590, and 2050 GHz/RIU with a figure of merit (FoM) of 3.20, 1.54, and 4.28 for the 1st, 2nd, and 3rd peaks, respectively. When the refractive index of the surrounding was fixed at 1.4, the results showed an ultra-sensitivity of 1230, 2270, and 1580 GHz/μm for the detection of thickness variation. The proposed design can be specifically useful for THz sensing applications.

## Figures and Tables

**Table 1:** The estimated parameters of the proposed design at optimum condition.

Parameter	Value (μm)
*P_x_*	8
*P_y_*	8
*d*	7.6
*L*	2
*l*	1.2
*t_r_*	0.03
*t_s_*	0.6
*t_g_*	0.03
*g*	0.5
w	0.3

**Table 2:** Comparison of the sensing performance between the proposed works with similar THz absorber-based sensor studies.

Reference	Techniques Used	Unit Cell Size $\left(\mu m^{2}\right)$	Substrate Thickness (μm)	Figure of Merit	Sensitivity (Analyte Change) GHz/µm	Sensitivity (Refractive Index Changing) GHz/RIU
Ref [30]	Au/Si/Au	50 × 50	3	1.39	153	-----
Ref [31]	Graphene/Dielectric/Au	6 × 6	2.7	4.21	-----	1687
Ref [32]	Graphene/Polyimide/Cu/Teflon	50 × 50	18	0.48	-----	22.75
Ref [33]	Metal/ polyimide	500 × 500	60	3	1.7	100
Ref [34]	Au/Dielectric/Au	36 × 36	10	2.94	23.7	300
Ref [35]	Metal/Dielectric/Metal	120 × 70	4.1	229.24	-----	1900
Ref [36]	Al/Polyimide/Si	150 × 150	18	2.67	-----	163
Ref [37]	metal split ring resonator/Si	70 × 70	16	19.35	-----	34
Ref [38]	Au/quartz/Au	300 × 300	20	1.50	7.64	-----
Ref [39]	Quartz/Al/Microfluidic channel/ Al/polyimide	96 × 96	2	5.31	-----	638
**This work**	**Metal/ZnSe/Metal**	**8 × 8**	**0.6**	**4.28**	**2270**	**2050**

## Data Availability

Not applicable.
